# Abscessed inguinal metastasis as an initial presentation of testicular cancer

**DOI:** 10.11604/pamj.2018.31.83.17049

**Published:** 2018-10-04

**Authors:** Carlos Eduardo Salazar-Mejía, Enrique González-Nava

**Affiliations:** 1 Centro Universitario Contra el Cáncer, University Hospital “Dr. José Eleuterio González” and Faculty of Medicine, Universidad Autónoma de Nuevo León, Monterrey, Nuevo León, México

**Keywords:** Testicular cancer, young men, inguinal metastasis, abscessed

## Image in medicine

A 24-year-old man presented to the emergency service with fever, malaise and a large abscessed tumor located in the left inguinal region.He had a history of hydrocele and varicocele treated surgically in childhood.One year before admission, a small nodule approximately 1 x 2 cm appeared in his left inguinal region; this nodule increased in size and was accompanied by a weight loss of approximately 15 kg. On arrival, a large abscessed and ulcered adenopathy 20 x 18 x 11 cm with purulent secretion was seen in the area associated with a 5 cm left testicular tumor. Imaging studies revealed a 1 cm left subpleural lung nodule on short axis. A radical left orchiectomy and a biopsy of the abscessed adenopathy were performed and the histopathologic report showed a mixed germ cell tumor. Tumor markers after orchiectomy were LDH 1124 IU/L, β-hCG 197mIU/mL, AFP 172 ng/mL. Wide spectrum antibiotics were started in addition to wide surgical debridement of the abscess with a favorable clincial response. After surveillance, the patient was discharged to receive ambulatory chemotherapy. Inguinal ganglionic metastasis associated with testicle cancer is a rare finding.The history of inguinal and/or scrotal surgery could explain this phenomenon as a result of an alteration of regional lymphatic drainage.When this group of patients develops testicular cancer, involvement of an inguinal node is described in up to 2-10% of cases.Despite this, routine inguinal lymphadenectomy is still controversial in this scenario.

**Figure 1:**
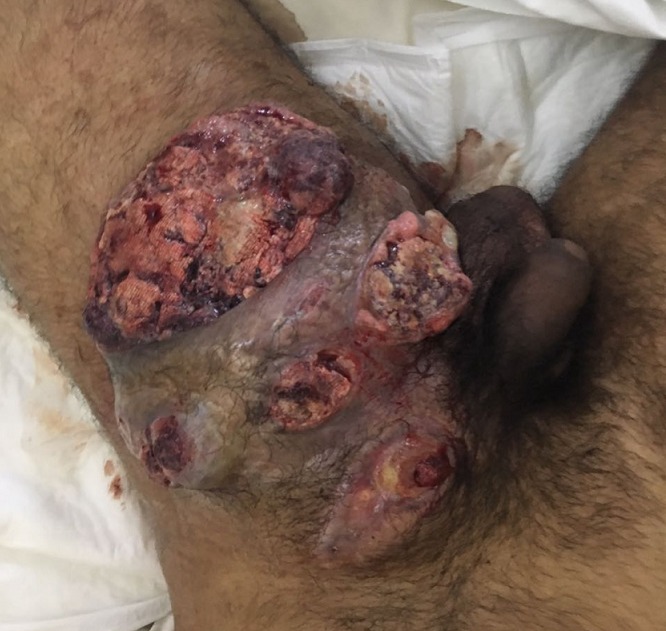
A large abscessed and ulcered adenopathy 20 x 18 x 11 cm in the left inguinal region

